# Predictors of traumatic birth experience among a group of Iranian primipara women: a cross sectional study

**DOI:** 10.1186/s12884-019-2333-4

**Published:** 2019-05-22

**Authors:** Solmaz Ghanbari-Homayi, Zahra Fardiazar, Shahla Meedya, Sakineh Mohammad-Alizadeh-Charandabi, Mohammad Asghari-Jafarabadi, Eesa Mohammadi, Mojgan Mirghafourvand

**Affiliations:** 1 Students’ Research Committee, Tabriz University of Medical sciences, Tabriz, Iran; 2 Women Reproductive Health Research Center, Tabriz University of Medical Sciences, Tabriz, Iran; 3 Member of South Asia Infant Feeding Research Network (SAIFRN), School of Nursing, Faculty of Science, Medicine and Health, University of Wollongong, Wollongong, Australia; 4 Social determinants of Health Research Center, Tabriz University of Medical sciences, Tabriz, Iran; 5 Department of Statistics and Epidemiology, Tabriz University of Medical Sciences, Tabriz, Iran; 6 Department of Nursing, School of Medicine, Tarbiat Modares University, Tehran, Iran

**Keywords:** Traumatic birth, Traumatic birth experience, Prevalence, Risk factors, Cross-sectional study

## Abstract

**Background:**

Traumatic birth experience has undesirable effects on the life of the mother, child, family, and society. The identification of predictive factors can be useful in improving birth experiences among women. This study aimed to assess the prevalence of a traumatic birth experience and identify its predictors among a group primiparous women.

**Methods:**

A cross-sectional study was conducted among 64 health centres in Tabriz, the second largest city in Iran. Cluster sampling was used to recruit 800 eligible women at one to 4 months postpartum. The Persian version of the Childbirth Experience Questionnaire was used to measure the womens’ birth experiences. Data were collected through face to face interviews and analysed mainly by multivariable logistic regression.

**Results:**

The prevalence of traumatic birth experience was 37% in the study group. The independent predictors of the traumatic birth experience were related to antenatal and intrapartum factors. The antenatal predictor was the lack of exercise during pregnancy (OR = 2.81, CI 1.40–5.63, *P* = .003) and the intrapartum predictors were the absence of pain relief during labour and birth (OR = 4.24, CI 2.12–8.50, *P* < .001), and the fear of childbirth (OR = 3.47, CI 1.68–7.19, P < .001).

**Conclusions:**

The findings revealed the high rate of traumatic birth experience among the primimarous women and identified the importance of a woman-centered care where a woman can actively make decision about the care she receives receive during labour and birth.

**Electronic supplementary material:**

The online version of this article (10.1186/s12884-019-2333-4) contains supplementary material, which is available to authorized users.

## Background

The majority of women have a positive perception about pregnancy and regard it as a potential source of power [1], however, during childbirth period some women develop a negative long-term memory about their experiences [2]. Although this memory can fade away with time, there is a risk of developing a long-term negative memory, even after 5 years [3]. The prevalence of traumatic birth experience varies in different countries. For example the prevalence of traumatic birth experience in Sweden is 7% [4], in Canada 9.3% [5] and in Atlanta 34% [6].

Traumatic birth experience may affect the future life of both the mother and child by causing lower self-esteem and poor mental health, Post-traumatic Stress Disorder (PTSD), poor maternal-neonatal attachment, avoidance of breastfeeding, and sexual malfunction [7–10]. Women with a traumatic birth experience are more likely to show acute stress reactions and postpartum depression [11, 12]. In a study of 1065 primiparous women, traumatic birth experience has been reported to increase the risk of poor self-rated health outcomes 2 months (OR = 1.4) and 1 year after birth (OR = 1.9) [13]. Traumatic birth experience not only affects women’s personal life, but also has undesirable impacts at a social level such as a tendency towards C-section or unwillingness of future childbearing [14, 15]. For example, in a study of 617 Swedish participants, 38% of women who had a traumatic birth experience did not have subsequent children, versus 17% of women with a positive experience [14].

Based on comprehensive non- Iranian studies, many factors can contribute to a traumatic birth experience. For instance, fear of childbirth [16, 17], pregnancy-related problems [18], previous traumatic birth experience, instrumental delivery [19], depression during pregnancy [16, 20], history of psychological problems [16, 19], lack of perceived support [21], older age [5], experience of violence or abuse [5, 17], perceived poor well-being, presence in prenatal classes, unwanted pregnancy [5], and neonatal admission to the Neonatal Intensive Care Unit (NICU) [5, 22] are known to be risk factors of traumatic birth experience. Regardless of these factors, traumatic birth experience is a complex mental concept and it is not related to only complicated birth outcomes. For example, a study of 1893 women reported positive experience when if they felt the caregivers try their best to provide good care even if they had unexpected or adverse birth outcomes [7].

Although birth experience is a global phenomenon, it can be influenced by women’s cultural, traditional and social background and values [23–25]. For example, in a qualitative study among a group of Nepalese women and their families, the degree a woman has control over birth, along with her preferences and expectations during delivery depends on her social values [25].

Although the prevalence of traumatic birth experience and its predictors has been studied in many Western countries, there is no Iranian study that has focused on this important matter.

## Methods

This is part of the first phase of a four-stage mixed method research project aiming at developing a new guideline to improve birth experience in Iranian primiparous women. The first phase was a cross-sectional study conducted on 800 primiparous women who had given birth in teaching, private, or organizational hospitals of Tabriz, the second largest city in Iran.

### Participants

The participants were recruited to the study over a period between one to four months after giving birth. The inclusion criteria were women older than 18 years with singleton, term, cephalic presentation and primiparous pregnancy. Since women’s previous birth experience could influence their current childbirth experience, we excluded multiparous women from the study. For the same reason, women with C-section and a history of depression including postpartum depression, neonatal death or any major neonatal abnormality were excluded from the study.

### Recruitment

Women who gave birth in teaching, private, and organizational hospitals of Tabriz were included. The samples were selected using the cluster sampling method. The urban and suburban health centers names in Tabriz (*n* = 114) were listed and numbered, and a total of 64 centers were randomly selected using the website of www.random.org. Then, the researcher refered to each center and the eligible women’s names in each center were listed and numbered, and the participants from mothers who had a birth in selected centers at one to four months postpartum were randomly selected based on quotas determined for each center using the website of www.random.org. After reviewing eligibility criteria, the study and its aims and objectives were explained to the potential participants. The women who consented to participate in the study were asked to attend their health centers on agreed dates to participate in a face to face interview. The participants were ensured about the matter of confidentiality.

### Data collection tool

Birth experience was measured by the Persian version of the Childbirth Experience Questionnaire version 2.0 (CEQ 2.0) (Additional file 1). The questionnaire consists of 23 questions, 20 of which are scored by likert scale (from 1 to 4 points) and 3 questions using the visual scale (from 0 to 100). Subscales of the CEQ 2.0 include own capacity (questions 1, 2, 4, 5, 6, 7, 21 and 22), professional support (questions 11, 13, 14, 15 and 16), participation (questions 8, 9, 10 and 12) and perceived safety (questions 3, 17, 18, 19, 20, and 23). Negative questions were scored in reverse order. The range of total score and subscales is between 1 and 4, and lower scores represent more traumatic experience. Validity and reliability of the Persian version of CEQ 2.0 has been verified with Cronbach’s alpha of 0.94. The psychometric properties of the Farsi version of the CEQ 2.0 will be reported in another paper. The reliability of the original questionnaire was also high among postpartum women (Cronbach’s alpha = 0.93) [26]. The traumatic birth experience was considered to be less than a standard deviation from the mean score of the population (mean score ≤ 2.50).

Socio-demographic, pregnancy, labour and birth information were collected using a checklist designed by the research team (Additional file 2). The validity of this checklist was confirmed through face and content validity, so that the questionnaire was distributed to ten experts and after collecting feedback from them, required modifications were made on the questionnaire. The questions included three main categores: a) socio-demographic data (age, education level, occupation, duration of marriage, marital satisfaction, household income, insurance status); b) antenatal data (history of abortion, planned pregnancy, attendance in prenatal classes, first source of support, doing exercise during pregnancy); and intrapartum data (gestational age, place of birth, length of stay in the labour room, permission to move or change position during labour, permission to choose the position of childbirth, augmentation, use of pharmacological or non-pharmaceutical methods to reduce pain, episiotomy, presence of companion and doula). For the purpose of the study, exercising during pregnancy was measured by a question with the response options of Yes or No. We asked about the frequency of any type of exercise in a week and the duration of each exercise. Normal walking and jogging were considered exercise.

### Sample size

A minimum sample size of 329 was calculated based on the formula of n = Z_α/2_^2^ *p*(1-p)/d^2^ and considering of *p* = 31% (prevalence of traumatic birth experience from a pilot study), d (precision) = 0.05 and Type I error rate of 5%. Due to the cluster sampling and 20% attrition rate, a sample size of 800 was considered as the final number.

### Ethical consideration

This project was approved by Ethics committee of Tabriz University of Medical Sciences (Ethical code: IR.TBZMED.REC.1396.786). All subjects signed the informed written consent form.

### Data analysis

Data were analyzed using SPSS version 21. Descriptive statistic was used to describe the socio-demographic, pregnancy, labour and childbirth characteristics and birth experience. The univariate logistic regression tests were used to test the correlation between socio-demographic, pregnancy, labour and childbirth variables with the traumatic birth experience. In the next step, variables with a relation of *p* < 0.1 in the univariate analysis were entered the multivariable logistic model for determining independent covariates. Four models were developed using multivariable logistic regression: a) using socio-demographic variables; b) antenatal variables; c) intrapartum variables; and d) all of the variables. Logistic regression results were reported as odds ratio (OR) with 95% confidence interval. All tests were two-tailed. *P*-value < 0.05 was considered statistically significant.

## Results

A total number of 800 women in Tabriz city (response rate: 84%) were enrolled in the study between 14 May 2018 and 16 November 2018. More than half of the women (66.3%) were aged 18 to 25 years old with a moderate economic status (67.8%). Nearly half of the mothers (44.4%) had high school education and only 19.3% had university education. The majority of mothers (94.3%) were housewives and satisfied with their marital life (95.8%). Nearly three-fourths of the women (73.6%) did not attend prenatal classes and did not attempt any exercise during pregnancy. While many women (65.5%) had to stay in the labour room for more than 12 h, only less than half of them were allowed to move during labour (40.1%), and a few could choose their childbirth positions (11.3%). Almost every woman had episiotomy (97.8%) and more than half had augmentation during labour. Only two women (0.25%) had a vacuum assisted delivery (operative birth). While many women stayed in the labour room for more than 12 h (Table 1).

**Table 1:** Socio-demographic, pregnancy, labour and birth characteristics among primiparous women (*n* = 800)

Age	N(%)	Husband’s age	N (%)	Economic status	N (%)
Socio-demographic					
18 to 25	533 (66.3)	18 to 25	192 (24.0)	Low	171 (21.4)
26–30	179 (22.4)	26–30	352 (44.0)	Middle	542 (67.8)
31 and above	88 (11.0)	31 and above	256 (32.0)	High	87 (10.9)
Education		Husband’s education		Marriage length	
High school and lower	355 (44.4)	High school and lower	356 (44.5)	5 year and below	723 (90.4)
Diploma	291 (36.4)	Diploma	294 (36.8)	6 year and above	77 (9.6)
University	154 (19.3)	University	150 (18.7)		
Work status		Husband work		Source of support	
House keeper	754 (94.3)	Unemployed	45 (5.7)	Husband	470 (58.8)
Employed	46 (5.7)	Employed	65 (8.2)	Mother or father	176 (22.0)
Self-employed	0 (0)	Self-employed	396 (49.5)	Relative	116 (14.5)
		Manual worker	294 (36.8)	Sister or brother	24 (3.0)
				Nobody	14 (1.8)
Insurance	470 (58.8)	Marital satisfaction	766 (95.8)		
Pregnancy					
Planned pregnancy	531 (66.4)	Prenatal class	222 (27.8)	Doing exercise during pregnancy	301 (37.6)
Abortion history		Pregnancy week		Duration of exercise (min)	
No	681 (85.1)	37 to 39	465 (58.1)	5 to 10	52 (17.2)
1 or above	119 (14.9)	40 and 41	335 (41.9)	15 to 30	195 (64.4)
				More than 30	56 (18.5)
Wanted pregnancy		Birth education class number (session)		Frequency of the exercise during one week	
No	105 (13.1)	1 or 2	81 (38.4)	1 or 2	85 (28.1)
Only me or my husband	35 (4.4)	3 or 4	44 (20.9)	3 or 4	89 (29.4)
Me and my husband	660 (82.5)	5 or 7	27 (12.8)	5 or 6	34 (11.2)
		8	59 (28.0)	7	95 (31.4)
Labour and childbirth					
Duration of stay in the labour room (Less than 12 h)	522 (65.5)	Doula presence	208 (26.0)	Baby sex (Girl)	421 (52.6)
Permission for moving in the labour room	479 (40.1)	Use of one of the pain relief methods	333 (41.6)	Presence of support person	191 (20.3)
Free in select of childbirth position	122 (11.3)	Augmentation	537 (67.1)	Fear of childbirth	514 (64.3)
Childbirth place		Episiotomy	782 (97.8)	Operative vaginal delivery	2 (0.25)
Organizational	113 (14.1)				
Public	555 (69.4)				
Private	132 (16.5)				

### Prevalence of traumatic birth experience

The mean of the total score of birth experience was 2.71. Out of the total 800 primiparous women, 37% of them had traumatic birth experiences (mean score ≤ 2.50) (Table 2). There was no statistically significant difference in the birth experience score between different times of completion of the questionnaire (1 month, 2 months, 3 months and 4 months postpartum) (*P* = 0.233).

**Table 2:** Birth experience and sub-scales score (n = 800)

Subscales	Mean (SD)	Number (%) traumatic score (≤2.50)
Own capacity	2.60 (0.79)	361 (45.1)
Participation	2.77 (0.82)	302 (37.8)
Perceived safety	2.67 (0.86)	347 (43.4)
Professional support	2.90 (0.92)	256 (32.0)
Total score	2.71 (0.73)	296 (37.0)

### Predictors of traumatic birth experience

There was a significant correlation between a traumatic birth experience with the following factors: socio-demographic factors (marital dissatisfaction, lack of insurance, inadequate household income, the first source of support other than husband), antenatal factors (unwanted pregnancy, not exercising during pregnancy), and intrapartum factors (childbirth in a teaching hospital, staying in the labour room for more than 12 h, no permission to move during labour, no permission to choose the position of childbirth, fear of childbirth, not using pain relief, including non-pharmacological and pharmacological methods, and the absence of a companion and doula) (*P* < 0.05) (Tables 3, 4 and 5).

**Table 3:** Correlation between socio-demographic factors with traumatic birth experience among primiparous women (n = 800)

Variables	n/N (%)	OR (95% CI)	P	Variables	n/N (%)	OR (95% CI)	P
Age (Years)				Job			
18 to 25 (Ref)	200/533 (37.5)	1		Employed (Ref)	6/13 (46.1)	1	
26–30	60/179 (33.5)	0.83 (0.58 to 1.20)	0.336	House keeper	290/787 (36.8)	0.68 (0.22 to 2.04)	0.493
31 and above	36/88 (37.5)	1.15 (0.72 to 1.82)	0.545				
Husband’s age (Years)				Husband’s job			
18 to 25 (Ref)	75/192 (39.0)	1		Employee (Ref)	19/65 (29.2)	1	
26–30	126/352 (35.7)	0.87 (0.60 to 1.25)	0.451	Unemployed	17/45 (37.7)	1.47 (0.65 to 3.28)	0.493
31 and above	95/256 (37.1)	0.92 (0.62 to 1.35)	0.673	Manual worker	111/294 (37.7)	1.46 (0.81 to 2.63)	0.349
				Self-employed	149/396 (37.6)	1.46 (0.82 to 2.58)	0.197
Marriage length (Years)				Marital satisfaction			
5 and below (Ref)	274/723 (37.9)	1		Yes (Ref)	276/766 (36.0)	1	
6 and above	22/77 (28.5)	0.65 (0.39 to 1.09)	0.109	No	20/34 (58.8)	2.53 (1.26 to 5.10)	0.009
Education level				Source of support			
Illiterate or elementary	30/87 (34.4)	1.00 (0.57 to 1.74)	0.992	Husband (Ref)	152/470 (32.3)	1	
Secondary or high school	98/268 (36.5)	1.09 (0.72 to 1.66)	0.657	Mother or father	81/176 (46.0)	1.78 (1.25 to 2.54)	0.001
Diploma	115/291 (39.5)	1.24 (0.82 to 1.87)	0.291	Sister or brother	51/116 (43.9)	1.64 (1.08 to 2.48)	0.019
Academic (Ref)	53/154 (34.4)	1		Friend	8/24 (33.3)	1.04 (0.43 to 2.50)	0.919
				Nobody	4/14 (28.5)	0.83 (0.25 to 2.71)	0.766
Husband’s education				Economic status			
Illiterate or elementary	38/93 (40.8)	1.46 (0.85 to 2.51)	0.161	High (Ref)	22/87 (25.2)	1	
Secondary or high school	108/263 (41.0)	1.48 (0.97 to 2.25)	0.068	Moderate	197/542 (36.3)	1.68 (1.00 to 2.82)	0.046
Diploma	102/294 (34.6)	1.12 (0.74 to 1.71)	0.570	Low	77/171 (45.0)	2.42 (1.36 to 4.27)	0.002
Academic (Ref)	48/150 (32.0)	1					
Insurance							
Yes (Ref)	153/470 (32.5)	1					
No	143/330 (43.3)	1.58 (1.18 to 2.11)	0.002

**Table 4:** Correlation between pregnancy factors with traumatic birth experience among primiparous women (n = 800)

Variables	n/N (%)	OR (95% CI)	P	Variables	n/N (%)	OR (95% CI)	P
Gestational age (Weeks)				Prenatal class attendance (Session)			
37 to 39 (Ref)	173/465 (37.2)	1		1 or 2	33/81 (40.7)	2.01 (0.96 to 4.20)	0.061
40 and 41	123/335 (36.7)	0.97 (0.73 to 1.31)	0.888	3 or 4	18/44 (40.9)	2.03 (0.87 to 4.70)	0.098
				5 or 7	7/27 (25.9)	1.02 (0.36 to 2.90)	0.960
				8 (Ref)	15/59 (25.4)	1	
Abortion history				Doing exercise during pregnancy			
No (Ref)	255/681 (37.4)	1		Yes (Ref)	81/301 (26.9)	1	
1 or above	41/119 (34.4)	0.87 (0.58 to 1.32)	0.533	No	215/296 (72.6)	2.05 (1.50 to 2.80)	< 0.001
Planned pregnancy				Kind of exercise			
Yes (Ref)	186/531 (35.0)	1		Walking (Ref)	59/234 (25.2)	1	
No	110/269 (40.8)	1.28 (0.94 to 1.73)	0.105	Pregnancy	20/63 (31.7)	1.38 (0.75 to 2.53)	0.299
				Other	2/6 (33.3)	1.48 (0.26 to 8.30)	0.654
Wanted pregnancy				Exercise number during week			
Me and my husband (Ref)	232/660 (35.1)	1		1 or 2	19/85 (22.3)	0.65 (0.33 to 1.28)	0.217
No	45/105 (42.8)	1.38 (0.91 to 2.10)	0.128	3 or 4	23/89 (25.8)	0.79 (0.41 to 1.51)	0.481
Only me or my husband	19/35 (54.2)	2.19 (1.10 to 4.34)	0.025	5 or 6	10/34 (29.4)	0.94 (0.40 to 2.23)	0.903
				7 (Ref)	29/95 (30.5)	1	
Time of exercise (min)				Presence in prenatal classes			
5 to 10	16/52 (30.7)	1.11 (0.48 to 2.53)	0.803	Yes (Ref)	76/222 (34.2)	1	
15 to 30	49/195 (25.1)	0.83 (0.43 to 1.63)	0.604	No	220/578 (38.0)	1.18 (0.85 to 1.63)	0.316
more than 30 (Ref)	16/56 (28.5)	1

**Table 5:** Correlation between labour and birth factors with traumatic birth experience among primiparous women (n = 800)

Variables	n/N (%)	OR (95% CI)	P	Variables	n/N (%)	OR (95% CI)	P
Duration of stay in the labour room (hour)				Augmentation			
Less than 12 (Ref)	178/522 (34.0)	1		No (Ref)	86/264 (32.5)	1	
12 and more than 12	117/275 (42.5)	1.43 (1.06 to 1.93)	0.019	Yes	210/537 (39.1)	1.33 (0.97 to 1.81)	0.069
Permission for moving during labour				Episiotomy			
Yes (Ref)	131/478 (27.4)	1		No (Ref)	6/18 (33.3)	1	
No	164/321 (51.0)	2.74 (2.04 to 3.69)	< 0.001	Yes	290/782 (37.0)	1.17 (0.43 to 3.17)	0.745
Free in select of childbirth position				Presence of companion			
Yes (Ref)	13/90 (14.4)	1		Yes (Ref)	29/162 (17.9)	1	
No	283/710 (39.8)	3.92 (2.14 to 7.20)	< 0.001	No	267/638 (41.8)	3.30 (2.14 to 5.08)	< 0.001
Fear of childbirth				Doula support			
No (Ref)	64/286 (22.3)	1		Yes (Ref)	46/207 (22.2)	1	
Yes	232/514 (45.1)	2.85 (2.05 to 3.96)	< 0.001	No	250/592 (42.2)	2.57 (1.78 to 3.71)	< 0.001
Use of one of the pain relief methods				Baby sex			
Yes (Ref)	73/333 (21.9)	1		Girl (Ref)	147/421 (34.9)	1	
No	223/467 (47.7)	3.25 (2.37 to 4.46)	< 0.001	Boy	149/379 (39.3)	0.82 (0.62 to 1.10)	0.199
Childbirth place (hospital)				Operative vaginal delivery			
Organizational (Ref)	25/113 (22.1)	1		No (Ref)	296/798 (37.0)	1	
Teaching	247/296 (83.4)	2.82 (1.75 to 4.53)	< 0.001	Yes	0/2 (0)	0.0 (0.0 to 0.0)	0.999
Private	24/296 (8.1)	0.78 (0.41 to 1.46)	0.443				

The results of multivariable logistic regression analysis showed that in the first model, using socio-demographic variables, lack of insurance, low level economic status, and the first source of support other than the husband were predictors of traumatic birth experience. The second model, using antenatal variables, showed that not doing exercise during pregnancy was a predictive factor. In the third model, using intrapartum variables, staying in the labour room for more than 12 h, no permission to move during labour, fear of childbirth, no use of one of the non-pharmacological or pharmacological methods for pain relief, childbirth in a teaching hospital, and augmentation were predictive factors. Finally, the results of the fourth model, using all variables, showed that the likelihood of having a traumatic birth experience was 2.81 times more among women who did not do any exercise during pregnancy [2.81 (1.40 to 5.63), *P* = 0.003]. Lack of use of pain relief during labour and having childbirth fear increased the likelihood of traumatic birth experience by 4.24 and 3.47 times, respectively [4.24 (2.12 to 8.50), *P* < 0.001], [3.47 (1.68 to 7.19), *P* = 0.001]. The Hosmer & Lemeshow test proposed that all of the models fit the data (*P* > 0.1). Socio- demographic, antenatal and intrapartum variables were responsible for five, nine and 25% of the total variance, respectively. However, considering all variables, traumatic birth experiences can be predicted by 45% (Table 6).

**Table 6:** Multivariable Regression Logistic model for the traumatic birth experience and influencing factors (n = 800)

Variables	OR (95% CI)	*P*
1. Demographic		
Marital satisfaction (Reference: Yes)		
No	1.92 (0.93 to 3.94)	0.075
Insurance (Reference: Yes)		
No	1.45 (1.07 to 1.97)	0.014
Economic status (reference: High)		
Low	1.90 (1.05 to 3.43)	0.033
Middle	1.53 (0.91 to 2.59)	0.106
Source of support (Reference: Husband)		
Mother or father	1.62 (1.13 to 2.33)	0.009
Relative	1.51 (0.99 to 2.31)	0.052
Sister or brother	1.02 (0.42 to 2.47)	0.954
Nobody	0.64 (0.19 to 2.16)	0.481
2. Pregnancy		
Doing exercise during pregnancy (Reference: Yes)		
No	3.19 (1.79 to 5.77)	< 0.001
3. Labour and childbirth		
Duration of stay in the labour room (Reference: Less than 12 h)		
More than 12 h	1.59 (1.13 to 2.24)	0.007
Permission for moving during labour (Reference: Yes)		
No	1.79 (1.27 to 2.52)	0.001
Free in select of childbirth position (Reference: Yes)		
No	1.79 (0.91 to 3.50)	0.087
Fear of childbirth (Reference: No)		
Yes	3.06 (2.15 to 4.36)	< 0.001
Use of one of the pain relief methods (reference: Yes)		
No	2.96 (2.07 to 4.23)	< 0.001
Augmentation (reference: No)		
Yes	1.49 (1.05 to 2.10)	0.022
Childbirth place (reference: Organizational)		
Teaching	1.72 (1.01 to 2.93)	0.043
Private	0.73 (0.37 to 1.43)	0.366
4. All variables		
Use of one of the pain relief methods (reference: Yes)		
No	4.24 (2.12 to 8.50)	< 0.001
Fear of childbirth (Reference: No)		
Yes	3.47 (1.68 to 7.19)	0.001
Doing exercise during pregnancy (Reference: Yes)		
No	2.81 (1.40 to 5.63)	0.003
Presence of companion (reference: Yes)		
No	3.30 (0.80 to 10.86)	0.108
Free in select of childbirth position (Reference: Yes)		
No	4.64 (0.94 to 2.81)	0.059

* Adjusted for all other demographic variables with a relation of *p* < 0.1 in the bivariate analysis. Variables of husband education, marriage length and income status were removed from the model. *P* = 0.934 for Hosmer & Lemeshow test of the goodness of fit, Nagelkerkes R^2^ = 0.052

* Adjusted for all other pregnancy variables with a relation of *p* < 0.1 in the bivariate analysis. Variables of gravida, planned pregnancy, wanted pregnancy and exercise number class were removed from the model. *P* = 0.623 for Hosmer & Lemeshow test of the goodness of fit, Nagelkerkes R2 = 0.095

* Adjusted for all other labour and childbirth variables with a relation of *p* < 0.1 in the bivariate analysis. Variable of midwife’s continuous presence was removed from the model. *P* = 0.969 for Hosmer & Lemeshow test of the goodness of fit, Nagelkerkes R2 = 0.254

* Adjusted for all other variables with a relation of p < 0.1 in the bivariate analysis. *P* = 0.490 for Hosmer & Lemeshow test of the goodness of fit, Nagelkerkes R2 = 0.448

## Discussion

This study was aiming to assess the prevalence of a traumatic birth experience and identify the predictors of a traumatic birth experience among primiparous women. The results of the study demonstrated a high rate of a traumatic birth experience (37%) which is consistent with the findings of a study conducted in an urban area in Atlanta, USA, in which 34% of women reported a traumatic birth experience [5]. In this American study, a childbirth was more considered as a gynecological procedure, painful and a dangerous circumstance which can lead to an extreme perception about pain and birth situation [5]. In contrast, traumatic childbirth experience prevalence has been reported much lower in other countries such as Sweden 7% [4], Netherlands 16% [27], and Norway 21.1% [17] where childbirth was considered as a normal event and women could choose to give birth at home [27, 28]. In an Iranian context, although childbirth is not considered a gynecology procedure, it is influenced heavily by medical interventions. For instance, in Iran performing episiotomy for primiparous women [29] and using oxytocin during labour are part of accepted and routine protocols. But in the countries like Sweden and Netherlands only less than half of the women receive episiotomy or oxytocin during labour and birth [4, 30, 31]. Routine use of oxytocin for augmentation during labour, performing episiotomy without women’s consent had been reported as obstetric violence [32] where women’s right is neglected by the health professionals [33]. However, woman centered care provides the freedom of choices and allowed women to actively involved in decision making. Woman-centred care is a care that is responsive to women’s requests, expectations and wishes [34].

The results of this study showed that no form of exercise during pregnancy is a predictor where the traumatic childbirth prevalence increased the odds of traumatic birth experience by 2.81 times. Evidence demonstrated that women who exercised during pregnancy reported a significantly lower level of pain than women who did not. Exercise probably helps pregnant women to maintain a good body condition and strong abdominal muscles for easy delivery. Women who exercise during pregnancy may be more satisfied of giving birth because of having lower levels of pain and higher self-efficacy [35]. A clinical trial study (add some more information about this clinical trial, such as where, who, how many) showed that women who used birthing balls during their third trimester had lower labour pain and higher self-efficacy during labour [35].

Results of our study showed that the probability of traumatic birth experience in the absence of any type of pain relief including non-pharmacological or pharmacological methods was 4.24 times more than using any type of pain relief methods. The results are consistent with a study in Netherlands that showed women who did not use pain relief during labour were 2.9 times more likely to have a traumatic birth experience [36]. The relationship between pain management and childbirth experience could be related to the fact that most women are aware of pain relief techniques and thus they will not have a positive birth experience if their pain relief expectations are unfulfilled [37, 38]. A systematic review of 13 studies, highlighted that women might expect to have labour without using pain relief, but they emphasized the importance of the availability of pain relief for the women during the childbirth [39].

Considering women’s cultural and social background, women’s pain perception can influence their childbirth experience. For example, in a qualitative study of 14 Swedish women, positive perception of labour pain was associated with less traumatizing experience even if they did not use epidural or any other type of pain relief [40]. However, when women perceive labour pain negatively, they may feel epidural as a medical intervention without the sense of control [4, 41, 42]. In Iranian culture, women perceive labour pain as a negative experiecne [43].

In our study, fear of childbirth increased the odds of traumatic birth experience by 3.47 times. Results of this study were consistent with the majority of relevant studies. In a study conducted in Norway, Henriksen et al. reported that the prevalence of traumatic birth experience was 5 times higher among women with fear of childbirth [17]. A severe fear of childbirth may cause psychological problems, such as anxiety, depression, and panic which, in turn, lead to a traumatic birth experience [44]. Women with severe fear of childbirth are typically unwilling to participate in preparation classes for pregnancy and standard cares, due to fear of embarrassment. Therefore, they may have a more traumatic birth experience because of inadequate knowledge [45].

### Strength and limitation

This study has a few strengths including the evaluation of the birth experience at one to 4 months postpartum which minimized the risk of false positive and unreal responses. The other strengths are about the multi-sited design of the study, the large sample size and a high response rate (84%) where the participants were selected randomly. The probable effect of social status on birth experience was also managed by selecting participants from both rural and urban areas that included different types of health care services such as public, teaching, private and ogranisational hospitals. Considering the above key strengths, the results of our study can be generalized to the entire local population and similar populations in different cities. Using interview for data collection was a weakness of this study. Women may not report events due to the sense of shame and embarrassment, specifically when they have a traumatic experience. To minimize this weakness, the interviews were conducted in a quiet and empty room only in the presence of the researcher and the participant. The participants were also ensured about anonymity and confidentiality. They were also ensured that the study would not affect the care services they receive. Although the exclusion of multiparous women or C-section were regarded as a strength of the study, those were also a weakness as the results could not be generalized to this group of women. Also, multiple testing analysis was another limitation.

## Conclusion and implications for practice

This study identified the high prevalence of a traumatic birth experience among Iranian primiparous women which is a warning bell for healthcare professionals. At the same time, identifying the predicting factors, assists maternity care managers, policy makers and caregivers to improve women’s birth experience. The main recommendations are a) to offer women different types of pain relief during labour and birth; b) to identify and consult women who have a fear of childbirth at early stages, and c) reinforce exercise during pregnancy. Overall, there is an urgent need for a woman-centred care where women can be actively involved in their care during labour and birth by choosing their desired pain relief, walking and moving around, accompanying favorite support person and final set themselves free of any childbirth fear.

## Additional files

Additional file 1: The Childbirth Experience Questionnaire – CEQ 2.0. (DOCX 112 kb)

Additional file 2: A) Socio-demographic checklist: B) Pregnancy checklist. C) Labour and birth checklist. (DOCX 19 kb)

## Abbreviations

CEQ-2: Childbirth Experiences Questionnaire version 2.0.
NICU: Neonatal Intensive Care Unit
PTSD: Post-Traumatic Stress Disorder

## References

[1] Thomson GM, Downe S (2010). Changing the future to change the past: women’s experiences of a positive birth following a traumatic birth experience. Journal of Reproductive and Infant Psychology.

[2] Simkin P (1991). Just another day in a woman's life? Women's long-term perceptions of their first birth experience. Part I. Birth.

[3] Waldenström U, Schytt E (2009). A longitudinal study of women’s memory of labour pain—from 2 months to 5 years after the birth. BJOG Int J Obstet Gynaecol.

[4] Waldenström U, Hildingsson I, Rubertsson C, Rådestad I (2004). A negative birth experience: prevalence and risk factors in a national sample. Birth.

[5] Smarandache A, Kim TH, Bohr Y, Tamim H (2016). Predictors of a negative labour and birth experience based on a national survey of Canadian women. BMC Pregnancy Childbirth.

[6] Soet JE, Brack GA, DiIorio C (2003). Prevalence and predictors of women’s experience of psychological trauma during childbirth. Birth.

[7] Garthus-Niegel S, Knoph C, von Soest T, Nielsen CS, Eberhard-Gran M (2014). The role of labour pain and overall birth experience in the development of posttraumatic stress symptoms: a longitudinal cohort study. Birth.

[8] Elmir R, Schmied V, Wilkes L, Jackson D (2010). Women's perceptions and experiences of a traumatic birth: a meta-ethnography. J Adv Nurs.

[9] Brown A, Jordan S (2013). Impact of birth complications on breastfeeding duration: an internet survey. J Adv Nurs.

[10] Ayers S, Eagle A, Waring H (2006). The effects of childbirth-related post-traumatic stress disorder on women and their relationships: a qualitative study. Psychol Health Med.

[11] Christl B, Reilly N, Smith M, Sims D, Chavasse F, Austin MP (2013). The mental health of mothers of unsettled infants: is there value in routine psychosocial assessment in this context?. Arch Womens Ment Health.

[12] Gürber S, Bielinski-Blattmann D, Lemola S, Jaussi C, von Wyl A, Surbek D (2012). Maternal mental health in the first 3-week postpartum: the impact of caregiver support and the subjective experience of childbirth–a longitudinal path model. J Psychosom Obstet Gynecol.

[13] Schytt E, Waldenström U (2007). Risk factors for poor self-rated health in women at 2 months and 1 year after childbirth. J Women's Health.

[14] Gottvall K, Waldenstrom U (2002). Does a traumatic birth experience have an impact on future reproduction?. BJOG..

[15] Storksen HT, Garthus-Niegel S, Adams SS, Vangen S, Eberhard-Gran M (2015). Fear of childbirth and elective caesarean section: a population-based study. BMC Pregnancy Childbirth.

[16] Ayers S, Bond R, Bertullies S, Wijma K (2016). The aetiology of post-traumatic stress following childbirth: a meta-analysis and theoretical framework. Psychol Med.

[17] Henriksen L, Grimsrud E, Schei B, Lukasse M (2017). Bidens study group. Factors related to a negative birth experience–a mixed methods study. Midwifery.

[18] Berg M, Lundgren I, Lindmark G (2003). Childbirth experience in women at high risk: is it improved by use of a birth plan?. J Perinat Educ.

[19] Andersen LB, Melvaer LB, Videbech P, Lamont RF, Joergensen JS (2012). Risk factors for developing post-traumatic stress disorder following childbirth: a systematic review. Acta Obstet Gynecol Scand.

[20] van Son M, Verkerk G, van der Hart O, Komproe I, Pop V (2005). Prenatal depression, mode of delivery and perinatal dissociation as predictors of postpartum posttraumatic stress: an empirical study. Clin Psychol Psychother: An International Journal of Theory & Practice.

[21] Tani F, Castagna V (2017). Maternal social support, quality of birth experience, and post-partum depression in primiparous women. J Matern Fetal Neonatal Med.

[22] Bryanton J, Gagnon AJ, Johnston C, Hatem M (2008). Predictors of women’s perceptions of the childbirth experience. J Obstet Gynecol Neonatal Nurs.

[23] Lupton D. Risk and sociocultural theory: new directions and perspectives. Cambridge University press, Cambridge, United Kingdom.

[24] Lewallen LP (2011). The importance of culture in childbearing. J Obstet Gynecol Neonatal Nurs.

[25] Kaphle S, Hancock H, Newman LA (2013). Childbirth traditions and cultural perceptions of safety in Nepal: critical spaces to ensure the survival of mothers and newborns in remote mountain villages. Midwifery.

[26] Dencker A, Taft C, Bergqvist L, Lilja H, Berg M (2010). Childbirth experience questionnaire (CEQ): development and evaluation of a multidimensional instrument. BMC Pregnancy and Childbirth.

[27] Rijnders M, Baston H, Schönbeck Y, Van Der Pal K, Prins M, Green J, Buitendijk S (2008). Perinatal factors related to negative or positive recall of birth experience in women 3 years postpartum in the Netherlands. Birth..

[28] EURO-PERISTAT Project with SCPE and EUROCAT. European Perinatal Health Report. The health and care of pregnant women and babies in Europe in 2010. May 2013. Available from: www.europeristat.com.

[29] Rasouli M, Keramat A, Khosravi A, Mohabatpour Z (2016). Prevalence and factors associated with episiotomy in Shahroud City, northeast of Iran. Int J Womens Health Reprod Sci.

[30] National Board of Health and Welfare. Official statistics of Sweden. Statistics – health and medical care. Pregnancies, deliveries and newborn infants. The Swedish Medical Birth Register. 2013:1973–2012.

[31] Bernitz S, Øian P, Rolland R, Sandvik L, Blix E (2014). Oxytocin and dystocia as risk factors for adverse birth outcomes: a cohort of low-risk nulliparous women. Midwifery..

[32] Jardim DMB, Modena CM (2018). Obstetric violence in the daily routine of care and its characteristics. Revista latino-americana de enfermagem.

[33] World Health Organization. The prevention and elimination of disrespect and abuse during facility-nbasedchidlbirth. Genebra:WHO;2014. http://apps.who.int/iris/bitstream/10665/134588/1/WHO_RHR_14.23_eng.pdf?ua=1&ua=1.

[34] Bohren MA, Hofmeyr GJ, Sakala C, Fukuzawa RK, Cuthbert A. Continuous support for women during childbirth. Cochrane Database Sys Rev. 2017;7. 10.1002/14651858.CD003766.pub6 PMC6483123 28681500

[35] Gau ML, Chang CY, Tian SH, Lin KC (2011). Effects of birth ball exercise on pain and self-efficacy during childbirth: a randomised controlled trial in Taiwan. Midwifery.

[36] Larsson C, Saltvedt S, Edman G, Wiklund I, Andolf E (2011). Factors independently related to a negative birth experience in first-time mothers. Sex Reprod Healthc.

[37] Kangas-Saarela T, Kangas-Kärki K (1994). Pain and pain relief in labour: parturients' experiences. Int J Obstet Anesth.

[38] Gibbins J, Thomson AM (2001). Women's expectations and experiences of childbirth. Midwifery.

[39] Lally JE, Murtagh MJ, Macphail S, Thomson R (2008). More in hope than expectation: a systematic review of women's expectations and experience of pain relief in labour. BMC Med.

[40] Lundgren I, Dahlberg K (1998). Women's experience of pain during childbirth. Midwifery.

[41] Hidaka R, Callister LC (2012). Giving birth with epidural analgesia: the experience of first-time mothers. J Perinat Educ.

[42] Lindholm A, Hildingsson I (2015). Women's preferences and received pain relief in childbirth–a prospective longitudinal study in a northern region of Sweden. Sexual & Reproductive Healthcare.

[43] Pazandeh F, Potrata B, Huss R, Hirst J, House A (2017). Women’s experiences of routine care during labour and childbirth and the influence of medicalisation: a qualitative study from Iran. Midwifery.

[44] Rouhe H, Salmela-Aro K, Gissler M, Halmesmäki E, Saisto T (2011). Mental health problems common in women with fear of childbirth. BJOG.

[45] Rouhe H, Salmela-Aro K, Toivanen R, Tokola M, Halmesmäki E, Ryding EL, Saisto T (2015). Group psychoeducation with relaxation for severe fear of childbirth improves maternal adjustment and childbirth experience–a randomised controlled trial. J Psychosom Obstet Gynecol.

