# Supplementary material for: Predictors of traumatic birth experience among a group of Iranian primipara women: a cross sectional study
Source: BMC Pregnancy Childbirth. 2019 May 22;19:182. doi: 10.1186/s12884-019-2333-4 (PMC6532129; doi:10.1186/s12884-019-2333-4)
Supplement: Supplementary file 1 — The Childbirth Experience Questionnaire – CEQ 2.0. (DOCX 112 kb) [file 12884_2019_2333_MOESM1_ESM.docx]

The Childbirth Experience Questionnaire – CEQ 2.0

Dear new mother,

One of the goals of childbirth care is to ensure a positive childbirth experience for the mother. The purpose of this questionnaire is to learn about how you experienced childbirth. Your answers, along with answers from other new mothers, will be used to evaluate childbirth care. It is important that you answer all the questions.

There are two ways to rate your experience, either by ticking a box or marking a line.

Examples:

Tick the box below the response choice that best corresponds to your opinion.

I eat fruit every day.

Totally agree

□

Mostly agree

⮽

Mostly disagree

□

Totally disagree

□

Indicate your opinion by marking on the line between the two end-points.

How much do you like apples?

X

Not at all My favorite fruit

*The questionnaire begins on the next page.*

*Thank you for participating and sharing your views.*

**1.** Labour and birth went as I had expected.

Totally agree

□

Mostly agree

□

Mostly disagree

□

Totally disagree

□

**2.** I felt strong during labour and birth.

Totally agree

□

Mostly agree

□

Mostly disagree

□

Totally disagree

□

**3.** I felt scared during labour and birth.

Totally agree

□

Mostly agree

□

Mostly disagree

□

Totally disagree

□

**4.** I felt capable during labour and birth.

Totally agree

□

Mostly agree

□

Mostly disagree

□

Totally disagree

□

**5.** I was tired during labour and birth.

Totally agree

□

Mostly agree

□

Mostly disagree

□

Totally disagree

□

**6.** I felt happy during labour and birth.

Totally agree

□

Mostly agree

□

Mostly disagree

□

Totally disagree

□

**7.** I felt that I handled the situation well.

Totally agree

□

Mostly agree

□

Mostly disagree

□

Totally disagree

□

**8.** I wish the staff had listened to me more during labour and birth.

Totally agree

□

Mostly agree

□

Mostly disagree

□

Totally disagree

□

**9.** I could get up and move around as much as I wanted.

Totally agree

□

Mostly agree

□

Mostly disagree

□

Totally disagree

□

**10.** I took part in decisions regarding my care and treatment as much as I wanted.

Totally agree

□

Mostly agree

□

Mostly disagree

□

Totally disagree

□

**11.** Both my partner and I were treated with warmth and respect.

Totally agree

□

Mostly agree

□

Mostly disagree

□

Totally disagree

□

**12.** I received the information I needed during labour and birth.

Totally agree

□

Mostly agree

□

Mostly disagree

□

Totally disagree

□

Totally agree

□

Mostly agree

□

Mostly disagree

□

Totally disagree

□

**14.** I would have preferred more encouragement from the midwife.

Totally agree

□

Mostly agree

□

Mostly disagree

□

Totally disagree

□

**15.** The midwife conveyed an atmosphere of calm.

Totally agree

□

Mostly agree

□

Mostly disagree

□

Totally disagree

□

**16.** The midwife helped me to find my inner strength.

Totally agree

□

Mostly agree

□

Mostly disagree

□

Totally disagree

□

**17.** My impression of the team’s medical skills made me feel secure.

Totally agree

□

Mostly agree

□

Mostly disagree

□

Totally disagree

□

**18.** I have many positive memories from childbirth.

Totally agree

□

Mostly agree

□

Mostly disagree

□

Totally disagree

□

**19.** I have many negative memories from childbirth.

Totally agree

□

Mostly agree

□

Mostly disagree

□

Totally disagree

□

Totally agree

□

Mostly agree

□

Mostly disagree

□

Totally disagree

□

**21.** As a whole, how painful did you feel childbirth was?

No pain Worst imaginable pain

**22.** As a whole, how much control did you feel you had during childbirth?

No control Complete control

**23.** As a whole, how secure did you feel during childbirth?

Not at all secure Completely secure

Additional comments:

Thank you for your input!
