# Supplementary material for: Predictors of traumatic birth experience among a group of Iranian primipara women: a cross sectional study
Source: BMC Pregnancy Childbirth. 2019 May 22;19:182. doi: 10.1186/s12884-019-2333-4 (PMC6532129; doi:10.1186/s12884-019-2333-4)
Supplement: Supplementary file 2 — A) Socio-demographic checklist: B) Pregnancy checklist. C) Labour and birth checklist. (DOCX 19 kb) [file 12884_2019_2333_MOESM2_ESM.docx]

1. **Socio-demographic checklist:**

Code: …

1. Health center name: …
2. Birth center name: …
3. Birth date: …
4. Husband’s birth date: …
5. Marriage age: …
6. Are you happy with your marriage? …
7. Are you satisfied with your marital life? Yes 🞏 No 🞏
8. Education: Illiterate🞏 elementary🞏 Secondary🞏 High school🞏 Diploma🞏 Academic🞏
9. Husband’s education: Illiterate🞏 elementary🞏 Secondary🞏 High school🞏 Diploma🞏 Academic🞏
10. Job: House keeper 🞏 Employed🞏
11. Husband’s job: Employee🞏 Unemployed🞏 Manual worker🞏 Self-employed🞏
12. How much does your monthly income suffice for living expenses?
13. It is completely enough 🞏 It is relatively enough 🞏 It is not enough 🞏
14. Are you covered by insurance? Yes 🞏 No 🞏
15. Who is your first source of support? Husband🞏 Mother or father🞏 Sister or brother🞏 Friend🞏 Nobody🞏 Other: …

B) **Pregnancy checklist:**

1. Date of delivery: ……
2. Gestational age (Weeks): …
3. Gravida: …
4. Abortion history: …
5. Had your pregnancy been planned? Yes 🞏 No 🞏
6. Had your pregnancy been wanted? No 🞏 Just I wanted🞏 Just my husband wanted 🞏 Both I and my husband wanted 🞏
7. Did you attend in prenatal class? Yes 🞏 No 🞏
8. If yes, How many sessions? …
9. Did you exercise during pregnancy? Yes 🞏 No 🞏
10. If yes, what kind of exercise? …
11. How many times during the week? …
12. Duration of exercise on average? …(min)

**C) Labour and birth checklist:**

1. Duration of stay in the labour room: … (hour)
2. Permission for moving during labour: Yes 🞏 No 🞏
3. Free in select of childbirth position: Yes 🞏 No 🞏
4. Fear of childbirth: Yes 🞏 No 🞏
5. Use of one of the pain relief methods: Yes 🞏 No 🞏
6. Augmentation: Yes 🞏 No 🞏
7. Episiotomy: Yes 🞏 No 🞏
8. Presence of companion: Yes 🞏 No 🞏
9. Doula support: Yes 🞏 No 🞏
10. Operative vaginal delivery: Yes 🞏 No 🞏
11. Baby sex: Girl 🞏 Boy 🞏
